# Supplementary material for: The Use of Off-Label Medications in Newborn Infants Despite an Approved Alternative Being Available—Results of a National Survey
Source: Pharmacy (Basel). 2022 Jan 25;10(1):19. doi: 10.3390/pharmacy10010019 (PMC8877519; doi:10.3390/pharmacy10010019)
Supplement: Supplementary file 1 [file pharmacy-10-00019-s001.zip › pharmacy-1531539-supplementary.pdf]

pharmacy MDPI

Supplemental Material: Survey Questionnaire (translated from German)

Dear esteemed colleagues!

In the context of a scientific project, we are conducting a nationwide survey in all Level 1 and Level 2 neonatal centers. We are interested to assess in what extend medications that are specifically approved for the neonatal population are used when a similar, off-label therapy that might be significantly cheaper is available.

Of course, your response and all data will be treated anonymously

Many thanks for your participation in this survey.

Please tick all that applies:

1. **For the treatment of a PDA, you use**

Ibuprofen (active ingredient Ibuprofen) □ or:

Pedea® (active ingredient Ibuprofen) □ or: ?

Number of PDA- treatments in your center

(in average during the last 5 years):

approx. 1- 50/year □ approx. 50-100/year □ > 100/Jyear □ no treatment of PDA □

2. For the treatment of neonatal apnea, you use

Coffein – Citrat (active ingredient Coffein- Citrat) □ or:

Peyona® (active ingredient Coffein- Citrat) □ or: ?

Number of neonatal apnea- treatments in your center

(in average during the last 5 years):

approx. 1- 50/year □ approx. 50-100/year □ > 100/Jyear □ no treatment of apnea □

3. For the treatment of infantile hemangiomas, you use

Propranolol (active ingredient Propranolol) □ or

Hemangiol® (active ingredient Propranolol) □ or: ?

Number of infantile hemangioma treatments in your center

(in average during the last 5 years):

approx. 1- 50/year □ approx. 50-100/year □ > 100/Jyear □ no treatment of hemangioma □
